# Pre-Clinical Evaluation of the Nanoliposomal antiPCSK9 Vaccine in Healthy Non-Human Primates

**DOI:** 10.3390/vaccines9070749

**Published:** 2021-07-06

**Authors:** Amir Abbas Momtazi-Borojeni, Mahmoud R. Jaafari, Maciej Banach, Armita Mahdavi Gorabi, Hedayat Sahraei, Amirhossein Sahebkar

**Affiliations:** 1 Nanotechnology Research Center, Pharmaceutical Technology Institute, Mashhad University of Medical Sciences, Mashhad 9177948564, Iran; abbasmomtazi@yahoo.com (A.A.M.-B.); jafarimr@mums.ac.ir (M.R.J.); 2 Department of Medical Biotechnology, School of Medicine, Alborz University of Medical Sciences, Karaj 3149969415, Iran; 3 Iran’s National Elites Foundation, Tehran 9311114578, Iran; 4 Biotechnology Research Center, Pharmaceutical Technology Institute, Mashhad University of Medical Sciences, Mashhad 9177948564, Iran; 5 Department of Preventive Cardiology and Lipidology, Medical University of Lodz, 93-338 Lodz, Poland; maciej.banach@icloud.com; 6 Cardiovascular Research Centre, University of Zielona Gora, 65-046 Zielona Gora, Poland; 7 Research Center for Advanced Technologies in Cardiovascular Medicine, Tehran Heart Center, Tehran University of Medical Sciences, Tehran 1411713138, Iran; armitamahdavi61@gmail.com; 8 Neuroscience Research Center, Baqiyatallah University of Medical Sciences, Tehran 9311114578, Iran; hsahraei1343@gmail.com; 9 Applied Biomedical Research Center, Mashhad University of Medical Sciences, Mashhad 9177948564, Iran; 10 School of Pharmacy, Mashhad University of Medical Sciences, Mashhad 9177948564, Iran

**Keywords:** nanoliposome, non-human primate, PCSK9, vaccine, cholesterol

## Abstract

Background: Our previous studies showed the safe preventive and therapeutic effects of immunization using the nanoliposomal antiPCSK9 vaccine called “Liposomal Immunogenic Fused PCSK9-Tetanus plus Alum adjuvant” (L-IFPTA), in mouse models of atherosclerosis. Here we aimed to ascertain the immunogenicity and safety of the L-IFPTA vaccine in a pre-clinical study in healthy non-human primates. Methods: Five male rhesus macaque monkeys were subcutaneously immunized with the L-IFPTA vaccine, four times with bi-weekly intervals. To evaluate immunogenicity, the plasma antiPCSK9 antibody in immunized monkeys was detected and quantified using the ELISA method. The functionality of the induced antiPCSK9 antibodies was determined by the PCSK9/LDLR in vitro binding assay kit. The safety of the vaccine was tested using the evaluation of several major circulating indicators including plasma lipid alterations, inflammatory biomarkers and organ injury biomarkers. Results: The resultant data indicated that the L-IFPTA vaccine significantly and highly induced the generation of functional and safe antiPCSK9 antibodies in immunized monkeys. Plasma levels of specific biomarkers indicating organ performance including creatinine, urea, uric acid, bilirubin, ALP, AS, ALT and TSH were not significantly altered. After immunization in healthy monkeys, non-prespecified endpoints (plasma levels of TC, LDL-C, VLDL-C and TG) were non-significantly reduced by 11.6 ± 36%; 16 ± 28%; 22 ± 53% and 24 ± 51%, respectively, while HDL-C was slightly increased by 2 ± 64%. There were also no significant changes in plasma levels of pro- and anti-inflammatory biomarkers. Conclusion: The L-IFPTA vaccine could efficiently stimulate the host humoral immune response to produce active antibodies that inhibit plasma PCSK9 while not provoking systemic inflammation and not adversely affecting organ performance.

## 1. Introduction

Currently, proprotein convertase subtilisin/kexin type 9 (PCSK9) inhibition is one of the most promising therapeutic approaches for lowering hypercholesterolemia and reducing the incidence of atherosclerotic cardiovascular disease (ACVD), particularly in patients with familial hypercholesterolemia and those experiencing statin resistance or intolerance [1,2,3,4]. PCSK9 is mainly produced by hepatocytes and secreted into the blood circulation where it can negatively regulate the protein expression of the low-density lipoprotein (LDL) receptor (LDLR) on the surface of hepatocytes. Circulating PCSK9 binds to the extracellular domain of LDLR, and then mediates the internalization and lysosomal degradation of LDLR, which leads to the lower clearance of LDL cholesterol (LDL-C) from the bloodstream and thus results in hypercholesterolemia [5].

Passive immunotherapy using monoclonal antibodies (mAbs) disrupting PCSK9/LDLR interaction provides the most advanced strategy for PCSK9 inhibition [6,7,8,9]. However, because of the short in vivo half-life of mAbs, their long-term therapeutic application needs frequent administration (once or twice/month) at relatively high doses that might not be affordable in many countries [10,11,12]. Moreover, the use of mAbs may be limited by tolerability problems and possible induction of host anti-mAb antibodies [11,12,13]. The active vaccination using PCSK9 peptide antigens has represented a desirable alternative strategy to overcome the drawbacks of mAbs. This strategy can provide the same therapeutic effects as those achieved with mAbs but with fewer injections and lower doses, and less risk of promoting drug-neutralizing immune responses.

The adjuvanted anti-PCSK9 vaccines have been studied by different approaches, including human recombinant PCSK9 with a DNA oligonucleotide adjuvant [14]; human PCSK9 peptides displayed on “virus-like particles” [15,16,17], PCSK9-BSA peptide [18]; and peptides mimicking PCSK9 epitopes conjugated to a KLH-carrier developed by AFFiRiS group using the AFFITOPE^®^ technology [19,20]. To date, only AFFiRiS group has tested the antiPCSK9 vaccine in a phase I human clinical trial; however, these results are not available yet.

During the recent few years, we have developed a new adjuvanted antiPCSK9 vaccine using a combination of the AFFITOPE^®^ and nanoliposome technology [21]. This vaccine, termed “Liposomal Immunogenic Fused PCSK9-Tetanus plus Alum adjuvant” (L-IFPTA) has been shown to have efficacy in various animal models [21,22,23,24]. The early study revealed that the L-IFPTA vaccine could effectively induce safe and long-term (one year) production of antiPCSK9 antibodies in BALB/c mice [21]. Further studies on C57BL/6 mice with severe hypercholesterolemia and atherosclerosis indicated that the L-IFPTA vaccine could efficiently exert long-lasting therapeutic [22] and preventive [25] effects against hypercholesterolemia and atherosclerosis. Such promising results of murine studies suggested L-IFPTA as an appropriate candidate for testing in clinical trials. To this end, in the present study, we aimed to ascertain the immunogenicity and safety of the L-IFPTA vaccine in healthy non-human primates, which have a high physiological similarity to humans.

## 2. Methods

### 2.1. The L-IFPTA Vaccine Formulation

The L-IFPTA vaccine was developed as explained in detail in our previous publication [21]. Briefly, to prepare L-IFPTA vaccine formulation, an immunogenic peptide construct embracing PCSK9 and tetanus epitopes (Table 1) termed “Immunogenic Fused PCSK9-Tetanus” (IFPT) was attached to the surface of already synthesized liposome nano-particles. Lipid formulation containing 1,2-Dimyristoyl-sn-glycero-3-phosphorylcholine (DMPC); 1,2-Dimyristoyl-sn-glycero-3-phosphorylglycerol (DMPG); and cholesterol (Avanti Polar Lipid; Alabaster, AL, USA) was particlized to homogeneous nanoliposomes, using the lipid-film hydration followed by the extrusion method. The IFPTA peptide construct (synthesized by ChinaPeptides Co., Ltd., Shanghai, China) was attached to the surface of the prepared nanoliposome particles (termed L-IFPT), using DSPE-PEG-Mal (1,2-distearoyl-*sn*-glycero-3-phosphoethanolamine-*N*-[maleimide(PEG)-2000]) lipid linker (Lipoid GmbH, Ludwigshafen, Germany). The conjugation between the peptides and liposome particles was confirmed using the thin layer chromatography (TLC); tricin-SDS-PAGE; and high-performance liquid chromatography (HPLC) as explained previously [21].

To purify peptide-conjugated liposome nanoparticles, the free peptides were eliminated using dialysis membrane sucks with 12–14 KD MWCO (Merck, Darmstadt, Germany; D6191-25EA).

Physical characters of L-IFPT nanoparticles, including particle size, surface charge, and polydispersity index (PDI) were measured using the dynamic light scattering (DLS) technique on a Zetasizer (Nano-ZS, Malvern, UK). To evaluate the stability, physical characters of L-IFPT nanoparticles stored at 4 °C and 25 °C have been measured monthly for up to six months. The vaccine formulation was completed by mixing the prepared L-IFPT nanoparticles to 0.4% Alum adjuvant (Sigma-Aldrich) at a 1:1 (*v*:*v*) ratio, which thereafter is called L-IFPTA.

### 2.2. Macaques Monkey Vaccination

**Figure 1:**
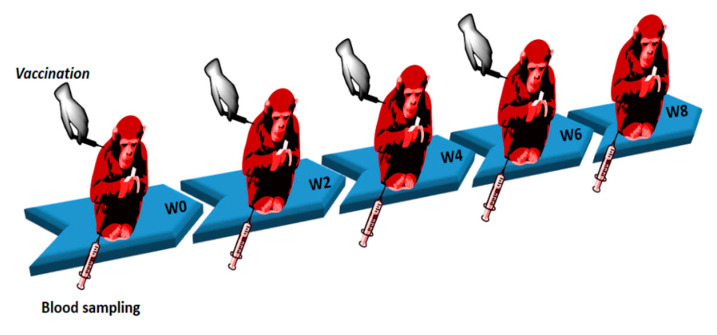
Schematic diagram of vaccination and blood sampling schedule.

Rhesus macaque monkeys (*Macaca mulatta*) were obtained from the animal house of the Neuroscience Research Center of the Baqiyatallah University of Medical Sciences (Tehran, Iran). All studies on macaques were performed by an expert veterinary physician in accordance with the animal welfare guidelines approved by the Institutional Ethics Committee and Research Advisory Committee of Mashhad University of Medical Sciences, Mashhad, Iran (IR.MUMS.PHARMACY.REC.1397.113). Five 7–9 year-old-male macaque monkeys (weight: 8–11 kg) were subcutaneously vaccinated four times at 2-week intervals with the L-IFPTA formulation containing 100 µg peptide per injection. Venous blood sampling was done prior to the vaccination at week 0 and two weeks following each vaccination up to week 8 (Figure 1). Redness, swelling or induration at the vaccination site, food intake and body weight, as well as malaise, fever, shivering or rash were checked during the vaccination period.

### 2.3. Characterization of Antibody Responses

To determine the immunogenicity of the L-IFPTA vaccine, titers of antiPCSK9-specific IgG were measured by ELISA technique, using PCSK9 peptide (ChinaPeptides Co., Ltd., Shanghai, China) as the antigen [21]. Detection was performed by HRP-conjugated anti-monkey IgG (Sigma Aldrich; dilution 1:1000), incubated for 1 h at 37 °C followed by the addition of the substrate TMB (3,3′,5,5′-tetramethylbenzidine) (Sigma-Aldrich; 15 min at RT). The optical density (OD) at 450 nm was measured with a microwell plate reader (BioTek, Synergy 2 plate reader, Winooski, VT, USA), and the titers were considered as the dilution factor corresponding to 50% of the maximal optical density (OD_max_/2).

### 2.4. Evaluating the Effect of Induced antiPCSK9 Antibodies on PCSK9/LDLR Interaction In Vitro

To evaluate the ability of vaccine-produced antibodies for hampering the PCSK9/LDLR interaction, an in vitro binding assay kit (CircuLex™ PCSK9/LDLR, Cy- 8150, MBL, Woburn, MA, USA) was used according to the manufacturer’s manual. The principle of the assay is based on the in vitro binding between recombinant PCSK9 and recombinant epidermal growth factor-like repeat (EGF-A) domain of LDLR, which would be suppressed in the presence of plasma containing antiPCSK9 antibodies. In this method, the higher amount of PCSK9 binding to LDLR is correlated with higher ELISA OD, in which at the presence of anti-PCSK9 antibody this interaction is suppressed, and, consequently, ELISA OD is reduced. The detailed protocol is clearly described in our previous publication [21].

### 2.5. The Plasma Lipid Assay

Plasma levels of total cholesterol (TC), VLDL-C, high-density lipoprotein cholesterol (HDL-C), and triglyceride (TG) in vaccinated monkeys were measured using the Biosystems kits (Biosystems S.A., Barcelona, Spain).

### 2.6. The Inflammatory Biomarker Assay

In order to assess systemic inflammation upon vaccination in macaque monkeys, a panel of plasma inflammatory markers, including L-1α, IL-1β, IL-2, IL-4, IL-6, IL-8, IL-10, VEGF, EGF, TNF-α, IFN-γ, hs-CRP, and MCP-1, were quantified using a cytokine array biochip (Randox, London, UK).

### 2.7. The Assessment of Organ Injury Biomarkers

As indicators for toxicity, plasma levels of biomarkers of organ injury, including creatinine, urea, uric acid, alkaline phosphatase (ALP), aspartate transaminase (AST), alanine transaminase (ALT), bilirubin, and thyroid-stimulating hormone (TSH) were measured in vaccinated macaque monkeys, using the Biosystems kits (Biosystems S.A., Barcelona, Spain) according to the manufacturer’s manual.

### 2.8. Statistical Analysis

An unpaired two-tailed Student’s *t*-test was carried out to define the significance of difference among groups (Graph Pad Prism Software, version 7, San Diego, CA, USA). Data were reported as mean ± SD. Values with *p* < 0.05 were regarded to be statistically significant.

## 3. Results

### 3.1. Characterization and Stability of L-IFPT Nanoparticles

**Figure 2:**
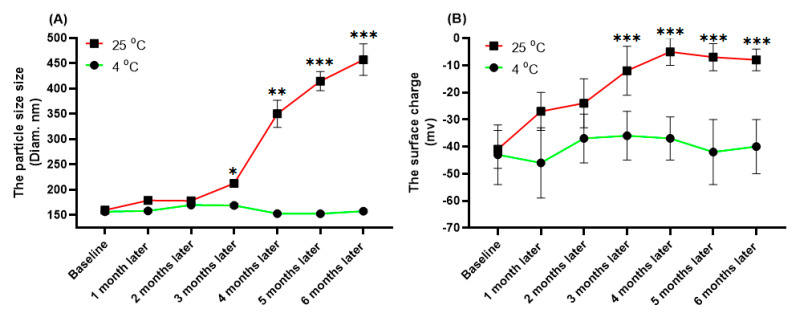
Evaluating the stability of L-IFPT nanoparticles by measuring changes of physical characteristics at 4 °C and 25 °C for 6 months. The physical characteristics of L-IFPT nanoparticles, including the size (**A**) and the surface charge (**B**), were not significantly changed for up to 6 months of storage at the 4 °C, while those were significantly increased after 3 months of storage at 25 °C, when compared to the baseline time point. Values are means ± SD (*n* = 3). Statistical differences at *p*-values less than 0.05 were considered to be significant. *, **, and *** signs show *p* = 0.03, *p* = 0.01, and *p* <0.01, respectively.

Physical properties of the free and IFPT-linked liposomal nanoparticles are summarized in Table 2. The size range of liposome nanoparticles was distributed from 130 nm to 160 nm in diameter, in which PDI was less than 0.01, indicating nanoparticles with high homogeneity. The prepared nanoliposomes also showed a negative surface charge. The physical properties of L-IFPT nanoparticles, including the size (Figure 2A) and the surface charge (Figure 2B), showed no significant changes after 6 months of storage at 4 °C, whereas significant changes were observed after 3 months at 25 °C, when compared to the baseline time point. Our results indicate that L-IFPT nanoparticles are stable for at least 6 months at the refrigerator temperature (4 °C) and for 3 months at room temperature (25 °C).

### 3.2. The L-IFPTA Vaccine Is Immunogenic in the Rhesus Macaque

**Figure 3:**
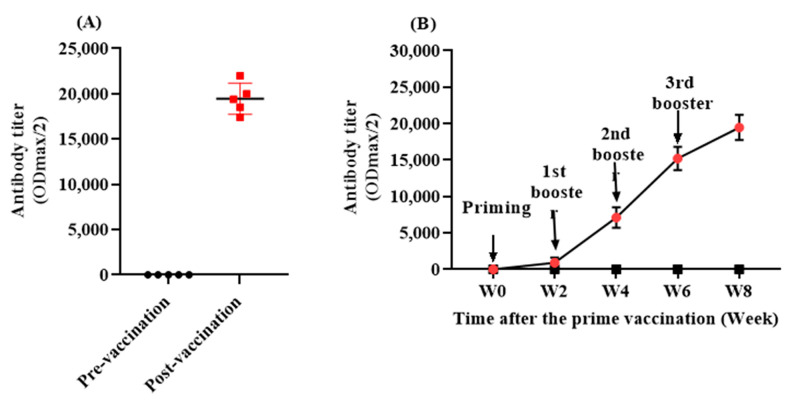
(**A**) Antibody titer (ODmax/2) against PCSK9 in vaccinated monkeys at pre-vaccination (W0) and post-vaccination (W8) time-points. (**B**) The exponential increase in antiPCSK9 antibody titer (ODmax/2) over 8 weeks post prime vaccination, generated upon 4 vaccinations in a biweekly interval (signed by arrows). Values are means ± SD (*n* = 5).

Titer analyses over time indicated that the L-IFPTA vaccine was able to significantly promote a humoral immune response against PCSK9 in rhesus macaque monkeys (Figure 3A). AnitPCSK9 IgG antibodies were significantly raised and reached a maximum level at the mean titer of 1: 19,460 ± 1726 (ODmax/2) at week 8, following three boosters after the prime immunization (Figure 3B).

### 3.3. Vaccine-Induced antiPCSK9 Antibodies Interfered with PCSK9 Function

**Figure 4:**
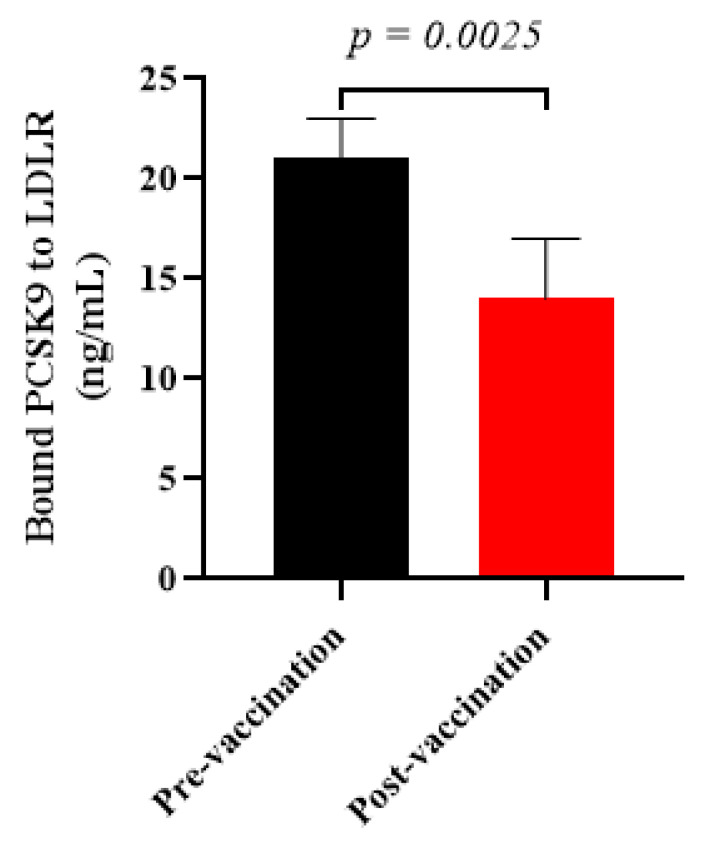
In vitro PCSK9/LDLR binding assay. Vaccine-produced antiPCSK9 antibodies suppress the interaction of PCSK9 and LDLR. A plasma sample of monkeys at post-vaccination time-point (W8) could reduce PCSK9 binding to LDLR by −33 ± 7%, when compared with a plasma sample of pre-vaccination ones (W0). Values are means ± SD; *n* = 3 replicates of the pooled samples of 5 monkeys. The significance compared to pre-vaccination values was analyzed by an unpaired two-tailed Student’s *t*-test. Statistical differences at *p*-values less than 0.05 were considered to be significant.

To evaluate whether the vaccine-induced antiPCSK9 antibodies are able to inhibit PCSK9 activity, the in vitro PCSK9/LDLR interaction was assayed in the presence of plasma samples isolated from vaccinated healthy monkeys at week 8. It was found that the post-vaccination plasma samples containing antiPCSK9 antibodies could significantly diminish PCSK9 binding to LDLR by −33 ± 7% when compared to pre-vaccination plasma samples (Figure 4). The resultant data indicated that L-IFPTA vaccine could provoke the production of antiPCSK9 antibodies in the rhesus macaques, which blocked the PCSK9 activity through targeting EGF-A binding domain, thereby, suppressing PCSK9-mediated LDLR degradation.

### 3.4. The L-IFPTA Vaccine Did Not Cause Organ Injury in the Rhesus Macaque

Plasma levels of specific indicators of the organ injury, including creatinine, urea, and uric acid (for kidneys); ALP, AST, ALT, and bilirubin (for the liver); as well as TSH (for thyroid glands), were not significantly changed in the vaccinated monkeys (Table 3).

### 3.5. The L-IFPTA Vaccine Did Not Change Levels of Plasma Lipids in the Rhesus Macaque

**Figure 5:**
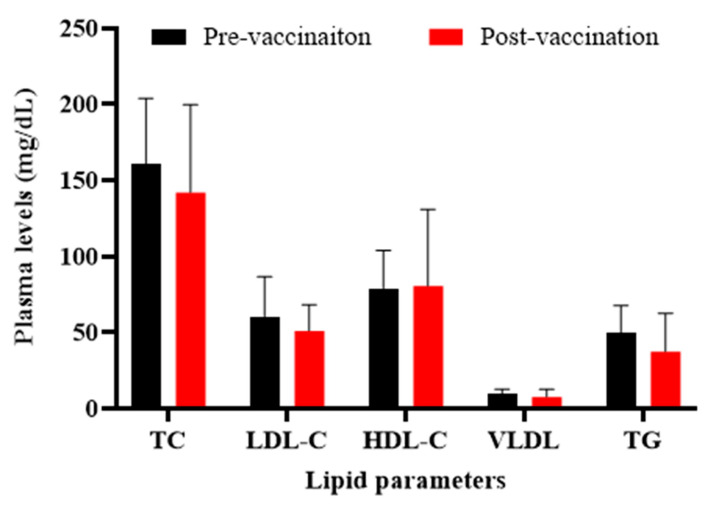
Plasma levels of lipid parameters in monkeys at pre- (W0) and post-vaccination (W0) time-points. There are no significant changes in plasma lipids levels, before and after vaccination, in the monkeys. Values are means ± SD (*n* = 5). The two-tailed Student’s *t*-test was used to evaluate the presence of significant differences between values of pre- and post-vaccination time-points. Statistical differences at *p*-values less than 0.05 were considered to be significant.

When compared the lipid profile in healthy monkeys at pre- and post-vaccination time-points, it was found that plasma levels of TC, LDL-C, VLDL-C, and TG were non-significantly (*p* > 0.05) decreased in vaccinated monkeys by 11.6 ± 36%, 16 ± 28%, 22 ± 53, and 24 ± 51%, respectively, while HDL-C was slightly increased by 2 ± 64% (*p* > 0.05) (Figure 5).

### 3.6. The L-IFPTA Vaccine Did Not Induce Systemic Inflammation in the Rhesus Macaque

The results demonstrated that the L-IFPTA vaccine does not have significant effects on inflammation-related biomarkers including IL-1α, IL-1β, IL-2, IL-4, IL-6, IL-8, IL-10, VEGF, EGF, TNF-α, IFN-γ, hs-CRP, and MCP-1 in vaccinated monkeys (Table 4).

## 4. Discussion

Inducing the host’s humoral immunity to produce functional and specific antibodies against a target molecule is an indispensable part of active immunotherapy. Here, we found that the L-IFPTA vaccine formulation could strongly induce the production of safe and functional antiPCSK9 antibodies in the rhesus macaques. Importantly, the vaccine-induced antibodies could significantly inhibit PCSK9 binding to the LDLR, confirming their functionality. This intervention was also safe, without any observed adverse effects.

These findings support our previous studies on BALB/c and C57BL/6 mice immunized by the L-IFPTA vaccine that showed similar results [21,22,26,27]. Of note, provoking antibody production in the two different species, rodents and non-human primates, is not surprising. This is because the immunogenic peptide antigen used in the formulation of the L-IFPTA vaccine [21] exploits AFFITOME^®^ technology that uses mimotope antigens with closely identical sequences between species and can thus show immunogenicity in various species [28].

Notably, despite the previously observed efficient therapeutic [22,26] and preventive [27] effects of the L-IFPTA vaccine for reducing hypercholesterolemia and atherosclerotic plaque progression in hyperlipidemic C57BL/6 mice, the present study showed a non-significant reduction in serum lipid indices in healthy monkeys. Of note, another anti-PCSK9 vaccine formulation prepared using virus-like particle technology was reported to decrease serum LDL-C in an approximately similar but significant percentage (by 10–15%) in healthy non-human primates [16]. Besides normolipidemic state, such a non-significant lipid reduction in the current study can be due to the small sample size of our study; therefore, the L-IFPTA vaccine might exert a significant and even a higher lipid-lowering effect with increasing the sample size and more suitable subjects (hyperlipidemic with additional risk factors, similarly to previous studies with PCSK9 inhibition [29]. The modest association between the effects of the vaccine on serum LDL-C (reduction by 16%) and the PCSK9 (inhibition by 33%) is not surprising, as it has been found that variation in levels of the circulating PCSK9 can explain only 7–8% of the variation in levels of circulating LDL-C [30]. It is also worth noting that the main aim of this study was to evaluate the immunogenicity and safety of the L-IFPTA vaccine in the healthy state and in a model with close physiological similarity to humans.

The adjuvanted vaccine formulations may be accompanied with increased local and systemic reactions called reactogenicity [31,32]. Reactogenicity signifies a subset of adverse reactions that appear soon after vaccination and is a physical manifestation of the inflammatory response to vaccination, including redness, swelling or induration at the vaccination site, as well as systemic symptoms, such as malaise, fever, shivering or rash [32]. In this study, we observed no such local and systemic symptoms in vaccinated monkeys.

It is suggested that the immune-mediated responses and injection-related reactions are the main mechanisms behind the progression of adverse events after vaccination. Vaccination may provoke the localized and systemic inflammatory responses including a destructive elevation in acute-phase proteins and proinflammatory cytokines [32]. The well-known inflammatory markers commonly employed for monitoring systemic inflammation responses after vaccination are TNF-α, IL-1, IL-6, and CRP [33]. At the injection site, the localized inflammatory response may be primed after vaccination due to the formation of antigen-antibody complexes and activation of the complement system, which rapidly induces inflammatory cells, such as tissue macrophages, to secrete pro-inflammatory cytokines, such as IL-1α, IL-1β, IL-6, TNF, and interferon, into the circulation, resulting in the systemic inflammatory response, such as fever [32,34]. Acting either directly or indirectly on the specialized neurons of the thermoregulatory center of the hypothalamus, these cytokines promote the generation of E-series prostaglandins that increase the host’s thermoregulatory set-point, leading to an elevation in the body temperature [34]. Moreover, systemic over-secretion of proinflammatory cytokines, together with reduced levels of anti-inflammatory cytokines, can result in systemic symptoms [34]. IL-1 and IL-6 have a central role in inducing the hepatic synthesis of the acute-phase proteins, such as CRP, in the liver. Notably, such inflammatory responses are mostly detectable in plasma or serum [33]. Our results indicated that the L-IFPTA vaccine exerted no significant effects on pro-inflammatory mediators (IL-1α, IL-1β, IL-6, TNF-α, IFN-γ, and hs-CRP) and anti-inflammatory cytokines (IL-4, IL-2, IL-8 and IL-10) in vaccinated monkeys. Furthermore, the MCP-1/VEGF/EGF pathway is involved in the migration and infiltration of inflammatory monocytes into the vascular wall and thereby provokes the local inflammation at the vaccination site [35,36,37]. We found no significant changes in the serum levels of such inflammatory mediators in the vaccinated monkeys.

The imbalanced production of inflammatory cytokines can result in tissue and organ damage [34]. Thus, the toxic effect on vital organs is another important concern about the safety of vaccines. Blood circulating biomarkers that manifest the damage of the liver, kidneys, and thyroid glands are considered as sensitive indicators for toxicity. Vaccinated monkeys showed no significant changes in ALP, AST, ALT, and bilirubin values as markers of liver function; creatinine, urea, and uric acid values as markers of kidney function; and TSH value as the marker of thyroid function. However, the results of toxicity studies, which are currently ongoing by the present research group, can also further determine the safety of the L-IFPTA vaccine in the near future. These findings are further supported by a recent study that showed the antiPCSK9 vaccine not only did not damage the liver and kidneys but also markedly alleviated the renal lipid deposition and fibrosis and reduced the formation of fatty liver in hypercholesterolemic mice [17]. Moreover, no effects on food intake, body weight, and mortality were found in vaccinated monkeys, further supporting the safety of the antiPCSK9 L-IFPTA vaccine.

## 5. Conclusions

In summary, our findings indicate that the L-IFPTA vaccine is sufficiently immunogenic and safe in non-human primates. Therefore, this pre-clinical result suggests that the L-IFPTA vaccine might be an eligible candidate for phase I clinical trials in humans.

## Figures and Tables

**Table 1:** Sequence of the immunogenic peptides used in the present study.

Peptide Name	Sequence	Immunogenicity
PCSK9	S-I-P-W-N-L-E-R-I-T-P-V-R	B cell epitope
Tetanus	A-Q-Y-I-K-A-N-S-K-F-I-G-I-T-E-L	T cell epitope
IFPT	* **CGGG**SIPWNLERITPVR**KK**AQYIKANSKFIGITEL	

* The bold amino acid codes exhibit linker sequences. IFPT; Immunogenic Fused PCSK9-Tetanus.

**Table 2:** Physical properties of nanoliposomal formulations.

Formulation	Z-Average (nm) [Mean ± SD, *n* = 3]	Zeta Potential (mV) [Mean ± SD, *n* = 3]	PDI * [Mean ± SD, *n* = 3]
The free-nanoliposome	134 ± 5	−41 ± 2	0.01 ± 0.001
The IFPT linked-nanoliposome	159 ± 8	−28 ± 3	0.02 ± 0.008

* PDI; Polydispersity index.

**Table 3:** Plasma levels of the organ injury indicators in monkeys at pre- and post-vaccination time-points.

Time-Point	Creatinine (mg/dL, Mean ± SD)	Urea (mg/dL, Mean ± SD)	Uric Acid (mg/dL, Mean ± SD)	Bilirubin (mg/dL, Mean ± SD)	ALP (mg/dL, Mean ± SD)	AST (mg/dL, Mean ± SD)	ALT (mg/dL, Mean ± SD)	TSH (mg/dL, Mean ± SD)
**Pre-immunization**	1.2 ± 0.2	30.8 ± 7	ND	ND	2311 ± 1121	32 ± 13	2.8 ± 1.5	0.9 ± 0.2
**Post-immunization**	1.1 ± 0.1	48 ± 14	ND	ND	1876 ± 1125	25 ± 14	2.4 ± 1.1	1 ± 0.3
**Mean difference**	NSD	NSD	-	-	NSD	NSD	NSD	NSD

Abbreviations: Alkaline phosphatase (ALP), Aspartate transaminase (AST), Alanine transaminase (ALT), Not defined (ND), NSD; No significant difference (NSD), Standard deviation (SD), Thyroid-stimulating hormone (TSH).

**Table 4:** Plasma levels of inflammatory biomarkers in monkeys at pre- and post-vaccination time-points.

Time-Point	IL1α	IL1β	IL2	IL4	IL6	IL8	IL10	IFNγ	TNF	EGF	VEGF	MCP-1	CRP
**Pre-vaccination** **(pg/mL, mean ± SD)**	ND	ND	ND	ND	1 ± 1.3	6.6 ± 8	3.6 ± 7	ND	ND	2.6 ± 6	ND	171 ± 31	2.2 ± 1.5
**Post-vaccination** **(pg/mL, mean ± SD)**	ND	ND	ND	ND	ND	25 ± 7	ND	ND	ND	62 ± 37	ND	156 ± 76	2 ± 0.7
**Mean difference**	-	-	-	-	NSD	NSD	NSD	-	-	NSD	-	NSD	NSD

IL: interleukin; IFN: interferon; TNF: tumor necrosis factor; EGF: epidermal growth factor; VEGF: vascular endothelial growth factor; MCP-1: monocyte chemoattractant protein-1; CRP: C-reactive protein; ND: not detected; NSD: no significant difference.

## Data Availability

Data associated with this study are available from the corresponding author on a reasonable request.
